# The classification of skateboarding tricks *via* transfer learning pipelines

**DOI:** 10.7717/peerj-cs.680

**Published:** 2021-08-18

**Authors:** Muhammad Amirul Abdullah, Muhammad Ar Rahim Ibrahim, Muhammad Nur Aiman Shapiee, Muhammad Aizzat Zakaria, Mohd Azraai Mohd Razman, Rabiu Muazu Musa, Noor Azuan Abu Osman, Anwar P.P. Abdul Majeed

**Affiliations:** 1 Innovative Manufacturing, Mechatronics and Sports Laboratory, Faculty of Manufacturing and Mechatronics Engineering Technology, Universiti Malaysia Pahang, Pekan, Pahang, Malaysia; 2 Centre for Fundamental and Liberal Education, Universiti Malaysia Terengganu, Kuala Nerus, Terengganu, Malaysia; 3 Department of Biomedical Engineering, Faculty of Engineering, Universiti Malaya, Kuala Lumpur, Kuala Lumpur, Malaysia; 4 Centre for Software Development & Integrated Computing, Universiti Malaysia Pahang, Pekan, Malaysia

**Keywords:** Classification, Support vector machine, Skateboarding, Machine learning, Transfer learning

## Abstract

This study aims at classifying flat ground tricks, namely Ollie, Kickflip, Shove-it, Nollie and Frontside 180, through the identification of significant input image transformation on different transfer learning models with optimized Support Vector Machine (SVM) classifier. A total of six amateur skateboarders (20 ± 7 years of age with at least 5.0 years of experience) executed five tricks for each type of trick repeatedly on a customized ORY skateboard (IMU sensor fused) on a cemented ground. From the IMU data, a total of six raw signals extracted. A total of two input image type, namely raw data (RAW) and Continous Wavelet Transform (CWT), as well as six transfer learning models from three different families along with grid-searched optimized SVM, were investigated towards its efficacy in classifying the skateboarding tricks. It was shown from the study that RAW and CWT input images on MobileNet, MobileNetV2 and ResNet101 transfer learning models demonstrated the best test accuracy at 100% on the test dataset. Nonetheless, by evaluating the computational time amongst the best models, it was established that the CWT-MobileNet-Optimized SVM pipeline was found to be the best. It could be concluded that the proposed method is able to facilitate the judges as well as coaches in identifying skateboarding tricks execution.

## Introduction

A skateboard is a short, narrow board with two small wheels attached to the bottom of either end. Skateboarders ride on this apparatus to perform tricks, including jumps (ollies), flips and mid-air spins. It is worth noting that this sport shall make its Olympic debut in the now delayed Tokyo 2020 Olympic Games. In general, in skateboarding competitions, the judging is done manually and subjectively through the observation of selected professional judges. However, it is worth mentioning at this juncture that the Head Judge for Skatepark of Tampa & Board has pointed out the myriad difficulties in providing a judgement in a skateboarding event (Pappalardo, 2014). Amongst the notable factors reported were the style, speed, difficulty, consistency, trick selection and originality. Such obstacles are also faced by the coaches in providing comprehensive feedback to further improve the performance of the athletes (Stein et al., 2018).

Owing to the advancement of technology, the employment of machine learning and, to a certain extent, deep learning has received due attention in human and sports activity recognition. For instance, Chen & Xue (2015) employed a deep learning model on data captured *via* an accelerometer for human activity recognition (HAR). The authors extracted the acceleration data through an android phone with a sampling frequency of 100 Hz from the built-in tri-axial accelerometer. Eight activities were investigated, *i.e*., falling, running, jumping, walking, step walking, walking quickly, walking downstairs and upstairs from 68 males and 32 females. A total of 31,688 labelled samples utilized, where 27,395 samples used for training, and the remaining 4,293 were used for testing. The authors fed the raw signal transformed images to Convolutional Neural Network with a three convolutional layer and three pooling layer architecture. Moreover, conventional feature extraction methods *via* Fast Fourier Transform, as well as Discrete Continuous Transform apart from the original time-domain signals that were paired with Support Vector Machine and Deep Belief Network models, were also investigated. It was shown from the study that the proposed CNN model could achieve a classification accuracy (CA) of 93.8% of accuracy and was better than that of the other models evaluated.

Akula, Shah & Ghosh (2018) investigated the use of multi-stage CNN on infrared images for HAR. The action data were collected from 18 females and 34 males between the age range of 19 to 28 years old. The FLIR E60 thermal infrared camera was utilized to capture a total of 5,278 image samples. The images consist of four main categories of actions, namely falling, sitting, walking, and standing. Additional subclasses for falling and sitting was also included, in which for falling includes fallen on the ground and fallen on the desk, whilst for sitting were sitting on a chair with and without a desk. The 5-fold cross-validation was employed. The images were split into training, validation, and testing phase with 28,844, 1,255, and 1,179 samples image, respectively. The proposed deep learning model could achieve a CA of 87.44% against the Histogram of Oriented Gradients (HOG)-SVM pipeline, which attained a CA of 85.9%.

Lee, Yoon and Cho (2017) evaluated the efficacy of CNN against a conventional machine learning model, *i.e*., Random Forest (RF), in the classification of HAR from data gathered *via* a tri-axial accelerometer. Five subjects participated in the study where three activities, namely staying still, running, and walking was recorded *via* Nexus 6P Huawei smartphones. The raw x, y and z signals were transformed into a single magnitude vector data (1D) with two-size feature vectors of 10 and 20 s denoted as Feature10 and Feature20, respectively. The RF model was evaluated *via* MATLAB whilst the CNN model *via* TensorFlow. It was shown from the study that the proposed 1D CNN achieved a CA of 91.32% for Feature10 and 92.71% for Feature20, respectively outperforming the conventional RF model, which achieved a CA of 85.72% and 89.10% for Feature10 and Feature20, respectively.

Conversely, Rangasamy et al. (2020) proposed the employment of the Transfer Learning paradigm for hockey activity recognition. The authors employed a pre-trained CNN, specifically VGG16, to extract features from four main hockey activities, namely free hit, goal, penalty corner and long corner, respectively. The dataset collected from International Hockey Federation (FIH) YouTube videos of the 2018 Hockey World Cup with a resolution of 1,280 $\times$ 720. A total of 400 frames been used and resized to 224 $\times$ 224 pixels. Different hyperparameters, namely the number of epochs with a different number of batch training of 100, 200 and 300, were fine-tuned at the fully connected layer utilizing a 10-fold cross-validation technique. The preliminary result showed that the model with 300 epochs achieved the highest CA of 98% then followed by 200 and 100 epochs with CA of 95% and 90%, respectively.

In relation to skateboarding, Groh, Kautz & Schuldhaus (2015) proposed the employment of machine learning in classifying six different skateboarding tricks using four machine learning classifiers, namely Naïve Bayes (NB), Partical Decision Tree (PART), Support Vector Machine with radial kernel basis kernel (RB-SVM) and k-Nearest Neighbor (kNN). Seven experienced male skateboarders between the age of 21 to 29 participated in the study. The data was gathered *via* an Inertial Measurement Unit (IMU) that was placed behind the front truck of the board. It was shown from the study that RB-SVM, as well as NB models, could achieve a CA of 97.8%. In an extended study, Groh et al. (2017) then enhanced the proposal by classifying thirteen classes for eleven skateboarding tricks, one class for bails and one class for other detected events with no trick. In this enhancement, the authors evaluated five classifiers which are NB, Random Forest (RF), Linear Support Vector Machine (LSVM), RB-SVM and kNN. It was shown from the study that the RB-SVM model was the best model amongst the models evaluated with a CA of 89.1%.

In a much earlier study, Anlauff et al. (2010) evaluated the efficacy of Linear Discriminat Analysis (LDA) in classifying three classes of two fundamental skateboarding tricks, *i.e*., Ollie and Ollie180 and one for no trick event. One skateboarder participated in the study, in which the skateboarder executed the tricks repeatedly for 20 times. A 10-fold cross-validation technique was employed on the training dataset, and it was shown from the study that an average CA of 89.33% was reported. Conversely, in a recent investigation, Corrêa et al. (2017) develop an Artificial Neural Network (ANN) model in classifying five skateboarding tricks. Interestingly, the authors artificially generated the dataset based on the acceleration data reported in Groh, Kautz & Schuldhaus (2015). A single hidden layer architecture was employed with 28 hidden neurons with a tan-sigmoid activation function trained with the Scaled Conjugate Gradient learning algorithm on a dataset that is split with an 80:20 ratio for training and validation. The study evaluated the model on data attained from the Z-axis only and the combination of XYZ axes. It was shown that the ANN developed for the Z-axis could achieve e a CA of 98.7%

In a more recent study, Abdullah et al. (2020) inspected six machine learning models, viz. SVM, kNN, ANN, Logistic Regression (LR), RF and NB in classifying five skateboarding tricks. An amateur skateboarder participated in the study in which the accelerometer and gyro data along the XYZ axes were acquired. Different statistical time-domain features were extracted from all the signals, *i.e*., mean, skewness, kurtosis, peak to peak, root mean square and standard deviation. A CA of 95% was reported to be attained *via* the features extracted on the LR and NB model. It could be seen from the limited literature available with regards to the employment of machine learning in classifying skateboarding tricks demonstrated commendable classification accuracy. Nevertheless, it is also evident from the literature reported that the use of CNN could mitigate the shortcomings of conventional machine learning models, particularly in acquiring significant features that would consequently yield better predictions. Therefore, this paper aims to address the gap by leveraging the use of a variation of the CNN model, *i.e*., the transfer learning model with its fully connected layer replaced with an optimized SVM model towards the classification of skateboarding tricks. The effect of input image transformation towards classification accuracy is also investigated.

## Methodology

### Data collection

**Figure 1:**
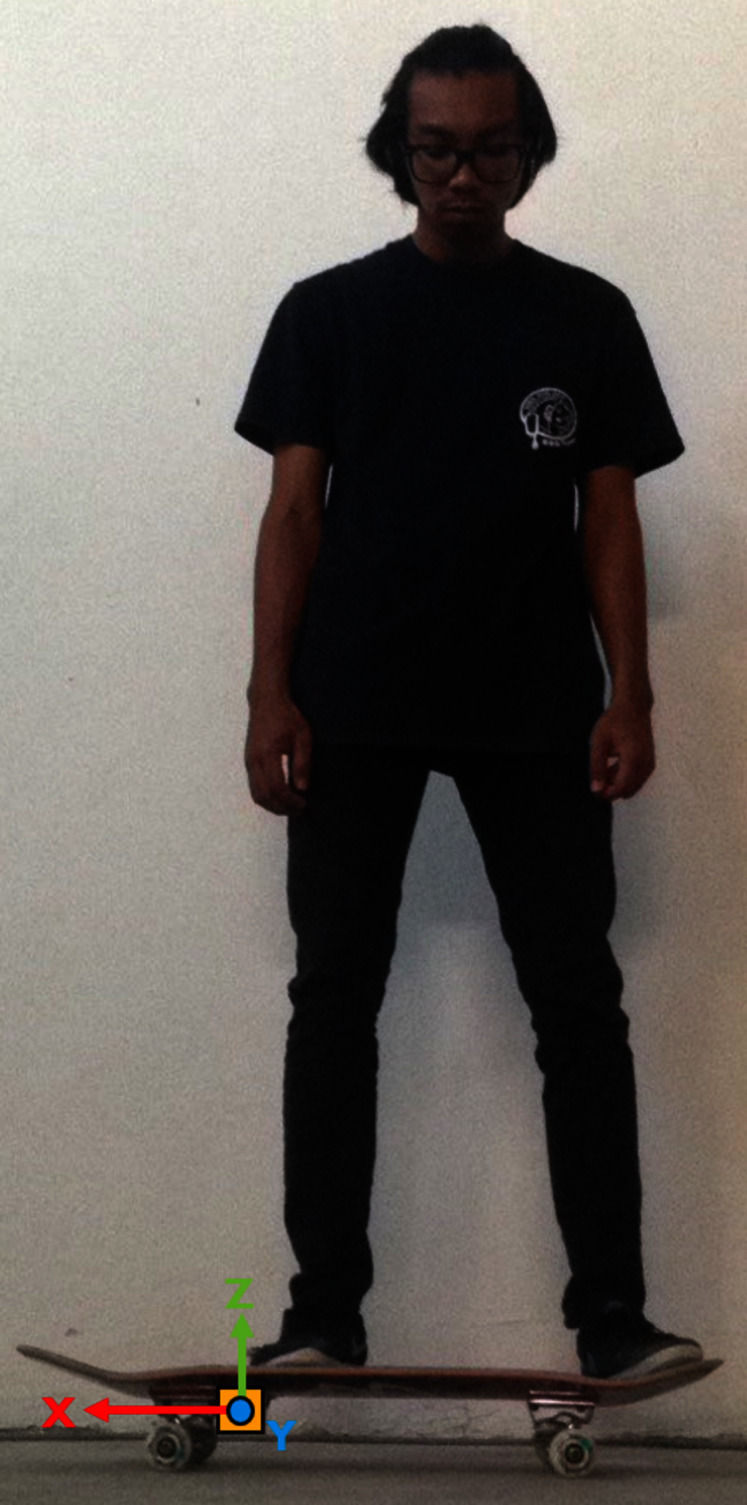
The position of the instrumented device on the skateboard (front view).

**Figure 2:**
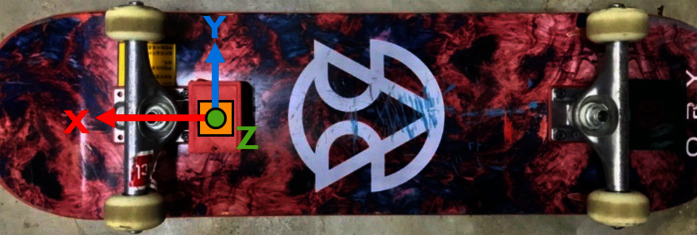
The placement of the instrumented device on the deck (rear view).

The skateboarding tricks signals were acquired through an instrumented inertial measurement unit (IMU) device developed. The device is embedded with an MPU6050 sensor, a Bluetooth 2.0 module, a microcontroller and a 3.7 V Lithium Polymer rechargeable battery. The device is paired together with a riser pad on the other side of the truck to give balance to the skateboard. Specifically, the device is mounted behind the front truck, and the pair are fixed with a nylon lock nuts (nyloc). The whole case and riser pad are made from ABS material printed *via* Zortrax M200 3D printer. The design of the IMU device is inspired by the works carried out by (Groh, Kautz & Schuldhaus, 2015). Figures 1 and 2 depicts the placement of the instrumented device from the frontal and rear view.

The chosen skateboarding tricks in the present investigation are Ollie (O), Nollie Frontside Shuvit (NFS), Frontside 180 (FS180), Pop Shove-It (PS) and Kickflip (KF). The selection of the tricks is non-trivial as it is the most common moves that are executed by a skateboarder in any competition (Groh, Kautz & Schuldhaus, 2015; Corrêa et al., 2017). The skateboarding tricks were performed by six 20 ± 7 years old amateur skateboarder with at least 5 years of experience and been executed successfully five times per trick. Universiti Malaysia Terengganu granted Ethical approval to carry out the study within and with its associated facilities (Ethical Application Ref: UMT/JKEPHMK/2021/53) whilst informed consent was obtained from the skateboarders participated in the present investigation.

### Input signal image transformation

In general, there were six simultaneous different raw signals data collected from the device per successful trick. The raw signals data were solely taken from the IMU embedded in the device. They are x-axis linear acceleration (aX), y-axis linear acceleration (aY), z-axis linear ac-celeration (aZ), x-axis angular acceleration (gX), y-axis acceleration (gX), and z-axis acceleration (gZ). All these six raw signals data were synthesized into a single image representing one single skateboarding trick according to the default image size based on Table 1.

**Table 1:** Default size settings of the transfer learning models.

No.	Model	Flatten reshape	Input image	
			Height	Width
1	MobileNet	7 * 7 * 1,024	224	224
2	MobileNetV2	7 * 7 * 1,280	224	224
3	NasNetLarge	11 * 11 * 4,032	331	331
4	NasNetMobile	7 * 7 * 1,056	224	224
5	ResNet101	7 * 7 * 2,048	224	224
6	ResNet101V2	7 * 7 * 2,048	224	224

Two input image transformation were chosen for this study. The basic input image was the raw transformation (RAW), where it is directly synthesized from the six raw signals stacked in a single image. The second input image transformation was a scalogram image transformed *via* Continuous Wavelet Transform (CWT). CWT is the representation of the time-frequency domain of a set of signals that have been demonstrated to be effective for non-stationary signals (Qassim et al., 2012). The resolution represented through the CWT algorithm has been reported to be beneficial owing to the exploitation of the small scale of high frequencies and large scale of low frequencies (Türk & Özerdem, 2019). Moreover, it has also been reported to provide a better representation of the arrangement of the frequency domain features as compared to Fourier Transforms. The mother wavelet that was used in this research is the Morlet Wavelet. Morlet wavelet is the multiplication of the complex exponential and Gaussian window. The Morlet algorithm gives an innate link between frequency and time domain to distinguish the signals acquired *via* Fourier Transform.

### Feature extraction: transfer learning

**Figure 3:**
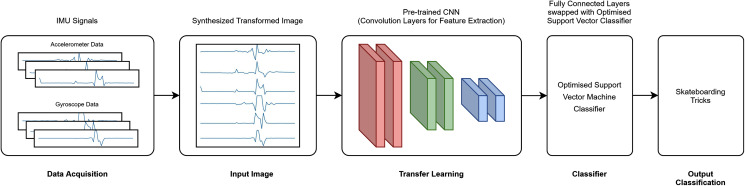
RAW-TL-optimized SVM pipeline.

**Figure 4:**
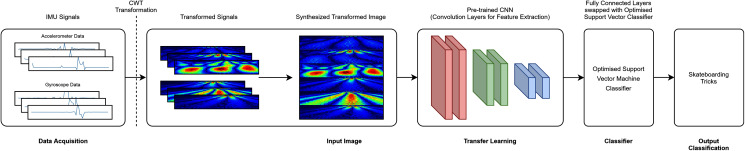
CWT-TL-optimized SVM pipeline.

A total of six transfer learning models was used for this study. The proposed architecture investigated is depicted in Fig. 3 (RAW) and Fig. 4 (CWT), respectively. This study exploits the use of three families of pre-trained CNN models, *i.e*., the MobileNet, NasNet and ResNet families. The rationale of employing transfer learning (TL) models is to reduce the model development time as the CNN models are not required to be built from scratch (Amanpour & Erfanian, 2013; Chronopoulou, Baziotis & Potamianos, 2019; Mahendra Kumar et al., 2021). A departure from conventional means of using such models is that the present study replaces the fully connected layers that is often referred to dense layers with a conventional machine learning model, *i.e*., SVM. Hence, the convolution layers of the transfer learning models utilized are used exclusively for feature extraction purpose. The list of the transfer learning model and their respective parameters are tabulated in Table 1.

### Classifier: support vector machine

The features extracted from the different transfer learning models based on the input images are fed into a variety of SVM models. The variation is based on the different hyperparameters evaluated, namely, the type of ***kernel***, viz linear, radial basis function (rbf) and polynomial (poly); the ***degree*** of the polynomial function, which was varied between two to six; (2–6); the kernel coefficient or ***gamma***, ***γ*** (0.1, 1, 10, 100); and strength of the regularization, ***C*** (0.01, 0.1, 1, 10, 100), respectively. It is worth noting that the ***γ*** parameter affects the rbf and poly-based SVM models. The loss function of the SVM classifier built-in in the scikit-learn package is the squared-hinge loss function. Table 2 lists the hyperparameters evaluated. The dataset was split into a ratio of 60:20:20 for training, testing and validation, respectively, on the 150 synthesized images per input transformation. The hyperparameters of the SVM models were tuned *via* the grid-search algorithm *via* the three-fold cross-validation technique on the training dataset. A total of 125 SVM models were developed per transfer learning model and per image input. Therefore, 1,500 pipelines (which consist of input image-transfer learning model-tuned SVM model) were evaluated in the present investigation. It is worth noting that the overall pipeline was evaluated on an Intel Core i7 4800MQ @ 2.70 GHz with 8 GB DDR3 800 MHz RAM and an Intel HD Graphics 4,600 *via* Spyder 3.3.6, a Python IDE running on Python 3.7 along with associated libraries, *i.e*., scikit-learn 0.22.1 and Keras 2.3.1: Tensorflow 1.14.0.

**Table 2:** Hyper-parameter description and range of value.

No.	Hyper-parameter	Description	Range
1	*Kernel*	Type of kernel	‘linear’, ‘poly’, ‘rbf’
2	*Degree*	Degree of polynomial function (only applicable for ‘poly’ kernel)	2–6
3	*Gamma, γ*	Kernel coefficient	0.1, 1, 10, 100
4	*C*	Strength of the regularization	0.01, 0.1, 1, 10, 100

Linear,

(1) $${y_i}\left( {w\cdot {x_i} + b} \right) \ge 1 - {\epsilon _i}\quad i = 1,\; \ldots ,\; m$$

Polynomial, *‘poly’*

(2) $$K\left( {x, x^{\prime}} \right) = {\left( {x\cdot {x + c}^\prime } \right)^q}$$

Radial basis function, *‘rbf’*

(3) $$K\left( {x, x^\prime } \right) = {e^{ - \displaystyle{{||x - \mathop x^\prime|| } \over {2{\sigma ^2}}}}}$$

where w is the weighting vector, b is the constant, and ε is the nonnegative slack variable.

### Performance evaluation

**Figure 5:**
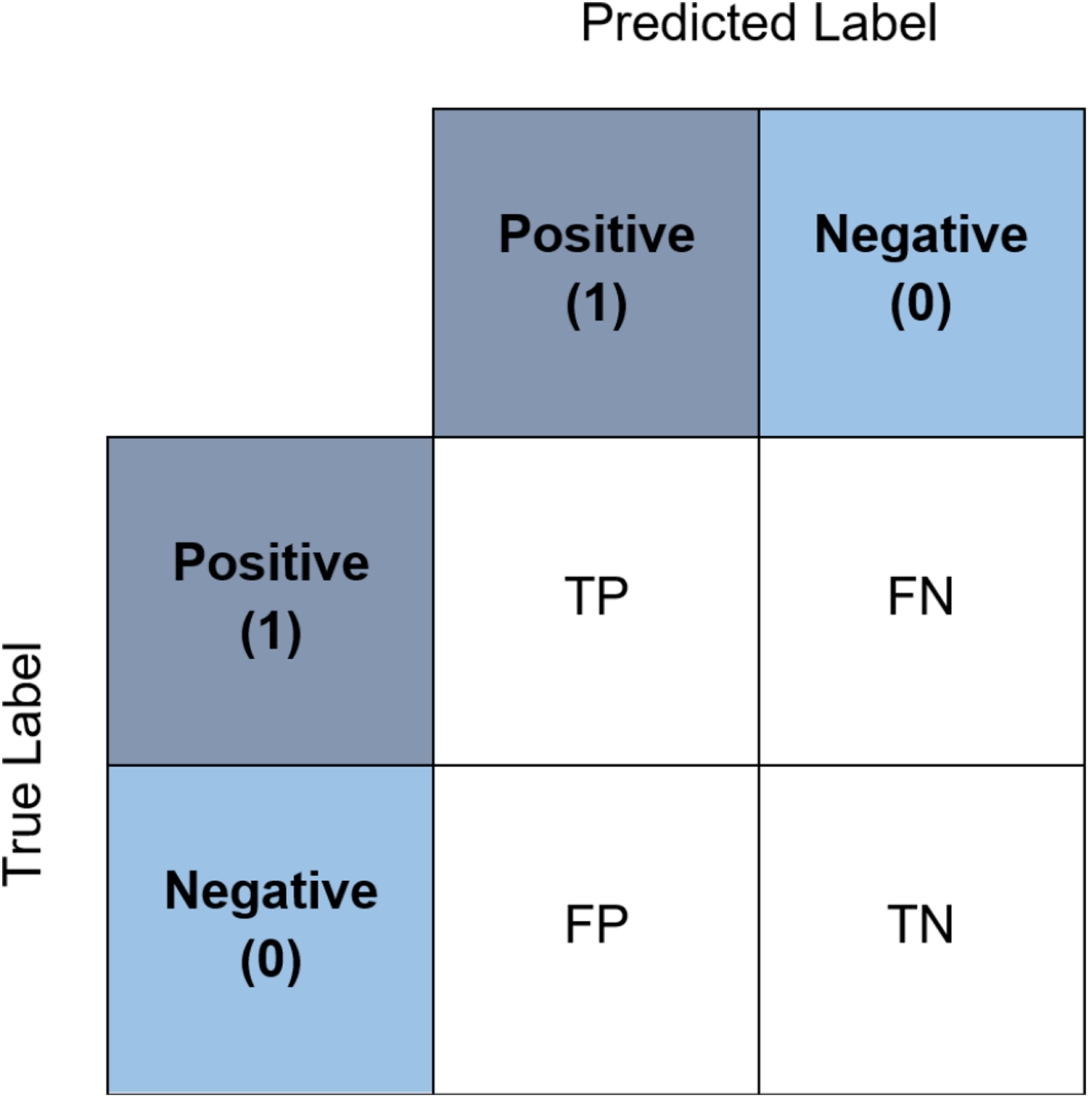
An example of a binary confusion matrix.

In the present study, a number of evaluation metrics were used. The accuracy score represents the accuracy of the model in predicting the corresponding value to the true value. The value ranges from zero to one where zero indicates a total misclassification transpired whilst one indicates that no misclassification transpired. It is commonly used to evaluate the accuracy of a multiclass classification problem (Foody & Mathur, 2004) and one of the most straightforward and simplest measures (Sokolova & Lapalme, 2009; Flach, 2019). This score also can be interpreted through the confusion matrix. Figure 5 illustrates an example of a binary class confusion matrix. True Positive (TP) is defined as a positive sample correctly predicted as positive. True Negative (TN) is the negative sample correctly predicted as negative. When the positive sample is incorrectly predicted as negative, it is counted toward the False Negative (FN). Conversely, False Positives (FP) is a negative sample incorrectly predicted as positive. The precision measures the percentage of correct positive predictions over the cumulative number of positive predictions. The sensitivity (often known as recall) is the number of true positive predictions divided by the sum of true positives as well as the false negatives (Vijay Anand & Shantha Selvakumari, 2019). The F1-score discloses the balance between the recall and the precision values. Whilst the specificity is essentially the proportion of actual negative values, which is forecasted as the true negative. It is also worth noting at this juncture, in the event that a tie between the classification accuracy transpire between the best pipelines, the determining factor would be based on the computational time of the pipelines.

## Experimental results and discussion

**Figure 6:**
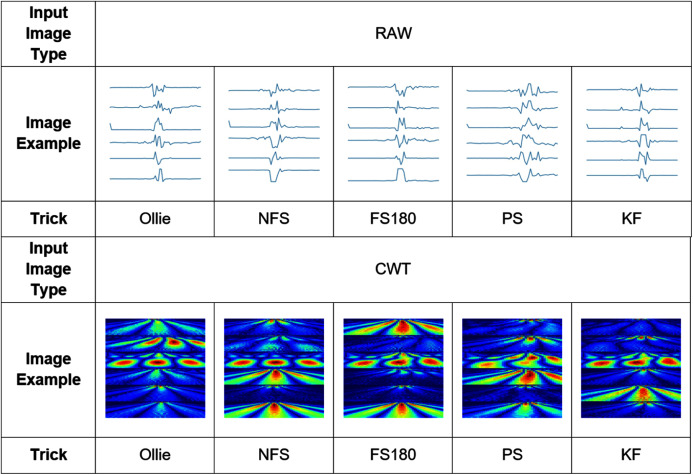
Example of the input image transformation.

**Figure 7:**
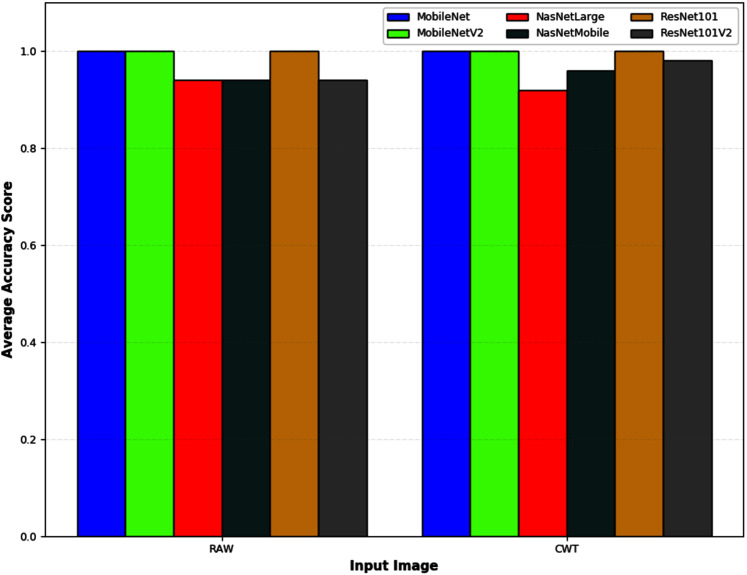
The average classification accuracy of different pipelines developed for the different input image evaluated.

**Figure 8:**
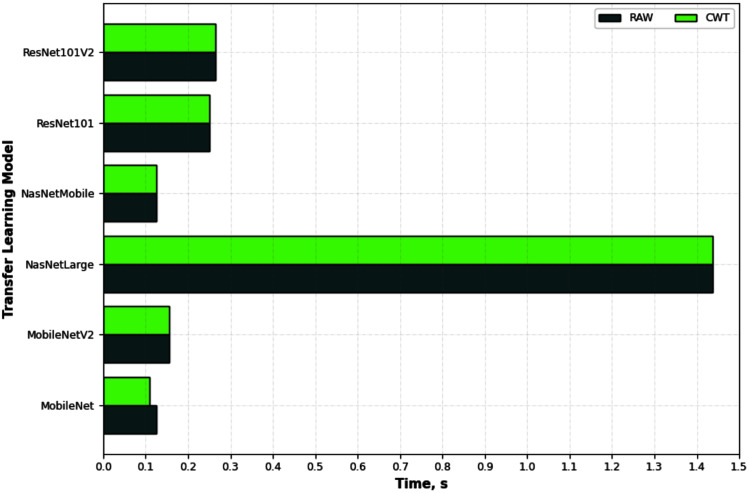
Prediction time of the different pipelines developed for the different input image evaluated.

Figure 6 depicts an example of the synthesized input images per skateboarding tricks with respect to RAW and CWT, respectively. Table 3 reports the accuracy of the evaluated pipelines. It could be observed that both RAW and CWT input transformation could yield an accuracy of 100% on all train, test and validation dataset for both MobileNet and ResNet101 families by utilizing the optimized SVM model. The optimized hyperparameters for the pipelines are the linear kernel-based SVM model with a C and gamma, ***γ*** value of 0.01 and 0.1, respectively. A similar performance is noticed for the RAW-ResNet101-optimized SVM as well as the CWT-ResNet101-optimized SVM models. Figure 7 depicts the average accuracy of the pipelines evaluated. Therefore, the determining factor for which pipeline is the best would be the computational time. As shown in Table 4, based on the computational time, the CWT-MobileNet-optimized SVM is deemed to be the best pipeline owing to the reduced computational time taken as compared to the other models evaluated. Figure 8 illustrates the aforesaid prediction time.

**Table 3:** Classification accuracy of transfer learning model with SVM for the different input image.

No.	Input image	Model	Accuracy			
			Train	Validate	Test	Average
1	RAW	MobileNet	1.00	1.00	1.00	1.00
2	RAW	MobileNetV2	1.00	1.00	1.00	1.00
3	RAW	NasNetLarge	1.00	0.88	1.00	0.94
4	RAW	NasNetMobile	1.00	0.88	1.00	0.94
5	RAW	ResNet101	1.00	1.00	1.00	1.00
6	RAW	ResNet101V2	1.00	0.88	1.00	0.94
7	CWT	MobileNet	1.00	1.00	1.00	1.00
8	CWT	MobileNetV2	1.00	1.00	1.00	1.00
9	CWT	NasNetLarge	1.00	0.88	0.96	0.92
10	CWT	NasNetMobile	1.00	1.00	0.92	0.96
11	CWT	ResNet101	1.00	1.00	1.00	1.00
12	CWT	ResNet101V2	1.00	1.00	0.96	0.98

**Table 4:** Computational time between the evaluated pipelines.

No.	Input image	Model	Time (s)		
			Train	Validate	Test
1	RAW	MobileNet	0.5000	0.1250	0.1250
2	RAW	MobileNetV2	0.1563	0.1563	0.1563
3	RAW	NasNetLarge	6.0000	1.5938	1.4375
4	RAW	NasNetMobile	0.4844	0.1250	0.1250
5	RAW	ResNet101	1.0063	0.2656	0.2500
6	RAW	ResNet101V2	1.0938	0.2653	0.2653
7	CWT	MobileNet	0.4688	0.1094	0.1094
8	CWT	MobileNetV2	0.6094	0.1563	0.1563
9	CWT	NasNetLarge	5.7188	1.4844	1.4375
10	CWT	NasNetMobile	0.5000	0.1406	0.1250
11	CWT	ResNet101	1.0156	0.2656	0.2500
12	CWT	ResNet101V2	1.0781	0.2656	0.2656

**Figure 9:**
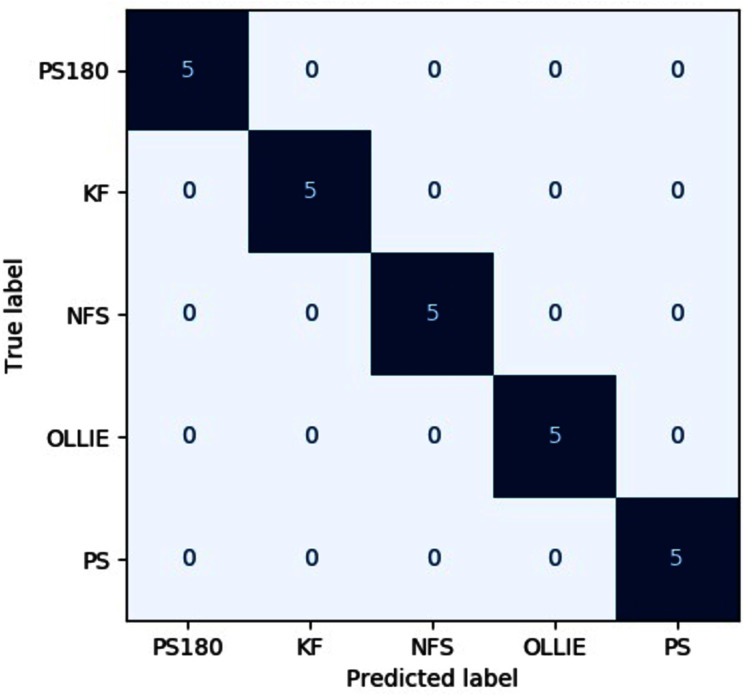
Confusion matrix of the best pipeline on the test dataset.

The precision, recall, F1-score, specificity on both input images across different evaluated pipelines are tabulated in Tables 5–9, respectively. The confusion matrix of the best pipeline, *i.e*., the CWT-MobileNet-optimized SVM on the test dataset, is depicted in Fig. 9. The present study has demonstrated that through the proposed pipeline, a better classification accuracy could be achieved as compared to the conventional means reported in the literature, particularly with regards to the classification of skateboarding tricks. The encouraging results reported suggests that the proposed pipeline could be beneficial in providing an objective-based judgment in The findings of the present investigation are in agreement with other studies that have employed such a technique in different applications, for instance, Lee, Yoon and Cho (2017), Rangasamy et al. (2020) as well as Mahendra Kumar et al. (2021). Nonetheless, it is worth noting that the efficacy of the pipelines is highly dependent on the dataset utilized, and the performance may vary. Future studies shall explore the use of different feature input transformation, other transfer learning as well as machine learning models.

**Table 5:** Precision of transfer learning model with SVM for the different input image.

No.	Input image	Model	Precision		
			Train	Validate	Test
1	RAW	MobileNet	1.00	1.00	1.00
2	RAW	MobileNetV2	1.00	1.00	1.00
3	RAW	NasNetLarge	1.00	0.89	1.00
4	RAW	NasNetMobile	1.00	0.93	1.00
5	RAW	ResNet101	1.00	1.00	1.00
6	RAW	ResNet101V2	1.00	0.93	1.00
7	CWT	MobileNet	1.00	1.00	1.00
8	CWT	MobileNetV2	1.00	1.00	1.00
9	CWT	NasNetLarge	1.00	0.91	0.97
10	CWT	NasNetMobile	1.00	1.00	0.93
11	CWT	ResNet101	1.00	1.00	1.00
12	CWT	ResNet101V2	1.00	1.00	0.97

**Table 6:** Recall of transfer learning model with SVM for different input image.

No.	Input image	Model	Recall		
			Train	Validate	Test
1	RAW	MobileNet	1.00	1.00	1.00
2	RAW	MobileNetV2	1.00	1.00	1.00
3	RAW	NasNetLarge	1.00	0.88	1.00
4	RAW	NasNetMobile	1.00	0.88	1.00
5	RAW	ResNet101	1.00	1.00	1.00
6	RAW	ResNet101V2	1.00	0.88	1.00
7	CWT	MobileNet	1.00	1.00	1.00
8	CWT	MobileNetV2	1.00	1.00	1.00
9	CWT	NasNetLarge	1.00	0.88	0.96
10	CWT	NasNetMobile	1.00	1.00	0.92
11	CWT	ResNet101	1.00	1.00	1.00
12	CWT	ResNet101V2	1.00	1.00	0.96

**Table 7:** F1-score of transfer learning model with SVM for different input image.

No.	Input image	Model	F1-score		
			Train	Validate	Test
1	RAW	MobileNet	1.00	1.00	1.00
2	RAW	MobileNetV2	1.00	1.00	1.00
3	RAW	NasNetLarge	1.00	0.88	1.00
4	RAW	NasNetMobile	1.00	0.87	1.00
5	RAW	ResNet101	1.00	1.00	1.00
6	RAW	ResNet101V2	1.00	0.87	1.00
7	CWT	MobileNet	1.00	1.00	1.00
8	CWT	MobileNetV2	1.00	1.00	1.00
9	CWT	NasNetLarge	1.00	0.86	0.96
10	CWT	NasNetMobile	1.00	1.00	0.92
11	CWT	ResNet101	1.00	1.00	1.00
12	CWT	ResNet101V2	1.00	1.00	0.96

**Table 8:** Specificity of transfer learning model with SVM for RAW input image.

No.	Input image	Model	Trick	Specificity		
				Train	Validate	Test
1	RAW	MobileNet	Ollie	1.00	1.00	1.00
			NFS	1.00	1.00	1.00
			FS180	1.00	1.00	1.00
			PS	1.00	1.00	1.00
			KF	1.00	1.00	1.00
2	RAW	MobileNetV2	Ollie	1.00	1.00	1.00
			NFS	1.00	1.00	1.00
			FS180	1.00	1.00	1.00
			PS	1.00	1.00	1.00
			KF	1.00	1.00	1.00
3	RAW	NasNetLarge	Ollie	1.00	0.95	1.00
			NFS	1.00	1.00	1.00
			FS180	1.00	0.95	1.00
			PS	1.00	0.95	1.00
			KF	1.00	1.00	1.00
4	RAW	NasNetMobile	Ollie	1.00	1.00	1.00
			NFS	1.00	1.00	1.00
			FS180	1.00	1.00	1.00
			PS	1.00	0.85	1.00
			KF	1.00	1.00	1.00
5	RAW	ResNet101	Ollie	1.00	1.00	1.00
			NFS	1.00	1.00	1.00
			FS180	1.00	1.00	1.00
			PS	1.00	1.00	1.00
			KF	1.00	1.00	1.00
6	RAW	ResNet101V2	Ollie	1.00	1.00	1.00
			NFS	1.00	1.00	1.00
			FS180	1.00	1.00	1.00
			PS	1.00	0.85	1.00
			KF	1.00	1.00	1.00

**Table 9:** Specificity of transfer learning model with SVM for CWT input image.

No.	Input image	Model	Trick	Specificity		
				Train	Validate	Test
1	CWT	MobileNet	Ollie	1.00	1.00	1.00
			NFS	1.00	1.00	1.00
			FS180	1.00	1.00	1.00
			PS	1.00	1.00	1.00
			KF	1.00	1.00	1.00
2	CWT	MobileNetV2	Ollie	1.00	1.00	1.00
			NFS	1.00	1.00	1.00
			FS180	1.00	1.00	1.00
			PS	1.00	1.00	1.00
			KF	1.00	1.00	1.00
3	CWT	NasNetLarge	Ollie	1.00	0.95	1.00
			NFS	1.00	1.00	0.95
			FS180	1.00	1.00	1.00
			PS	1.00	0.90	1.00
			KF	1.00	1.00	1.00
4	CWT	NasNetMobile	Ollie	1.00	1.00	1.00
			NFS	1.00	1.00	0.95
			FS180	1.00	1.00	0.95
			PS	1.00	1.00	1.00
			KF	1.00	1.00	1.00
5	CWT	ResNet101	Ollie	1.00	1.00	1.00
			NFS	1.00	1.00	1.00
			FS180	1.00	1.00	1.00
			PS	1.00	1.00	1.00
			KF	1.00	1.00	1.00
6	CWT	ResNet101V2	Ollie	1.00	1.00	1.00
			NFS	1.00	1.00	0.95
			FS180	1.00	1.00	1.00
			PS	1.00	1.00	1.00
			KF	1.00	1.00	1.00

## Conclusion

The present study investigated the efficacy of different transfer learning pipeline towards the classification of skateboarding tricks. It was shown that the study that best pipeline identified is the CWT-MobileNet-optimized SVM as it could yield the fastest computational time. It could be seen that the convolution part of the pre-trained CNN models or transfer learning models could effortlessly extract significant features, regardless of the input image provided. The findings are non-trivial in the realization of an objective-based judgement in a skateboarding competition. Future studies shall evaluate other types of input image transformation methods and transfer learning models as well as their effect towards other classifiers that have yet been investigated in the present study.

## Supplemental Information

10.7717/peerj-cs.680/supp-1 Supplemental Information 1 Code.

10.7717/peerj-cs.680/supp-2 Supplemental Information 2 Raw Dataset.

## Additional Information and Declarations

### Data Availability

The following information was supplied regarding data availability: Raw data and sample code are available in the Supplemental Files.

## References

[ref-1] Abdullah MA, Ibrahim MAR, Shapiee MNAB, Mohd Razman MNA, Musa RM, Abdul Majeed APP (2020). The classification of skateboarding trick manoeuvres through the integration of IMU and machine learning. Lecture Notes in Mechanical Engineering.

[ref-2] Akula A, Shah AK, Ghosh R (2018). ScienceDirect deep learning approach for human action recognition in infrared images. Cognitive Systems Research.

[ref-3] Amanpour B, Erfanian A (2013). Classification of brain signals associated with imagination of hand grasping, opening and reaching by means of wavelet-based common spatial pattern and mutual information.

[ref-4] Anlauff J, Weitnauer E, Lehnhardt A, Schirmer S, Zehe S, Tonekaboni K (2010). A method for outdoor skateboarding video games.

[ref-5] Chen Y, Xue Y (2015). A deep learning approach to human activity recognition based on single accelerometer.

[ref-6] Chronopoulou A, Baziotis C, Potamianos A (2019). An embarrassingly simple approach for transfer learning from pretrained language models. Proceedings of the 2019 Conference of the North American Chapter of the Association for Computational Linguistics: Human Language Technologies.

[ref-7] Corrêa NK, de Lima JCM, Russomano T, dos Santos MA (2017). Development of a skateboarding trick classifier using accelerometry and machine learning. Research on Biomedical Engineering.

[ref-8] Flach P (2019). Performance evaluation in machine learning: the good, the bad, the ugly, and the way forward. Proceedings of the AAAI Conference on Artificial Intelligence.

[ref-9] Foody GM, Mathur A (2004). A relative evaluation of multiclass image classification by support vector machines. IEEE Transactions on Geoscience and Remote Sensing.

[ref-10] Groh BH, Fleckenstein M, Kautz T, Eskofier BM (2017). Classification and visualization of skateboard tricks using wearable sensors. Pervasive and Mobile Computing.

[ref-11] Groh BH, Kautz T, Schuldhaus D (2015). IMU-based trick classification in Skateboarding.

[ref-12] Lee SM, Yoon SM, Cho H (2017). Human activity recognition from accelerometer data using convolutional neural network.

[ref-13] Mahendra Kumar JL, Rashid M, Muazu Musa R, Mohd Razman MA, Sulaiman N, Jailani R, Abdul Majeed APP (2021). The classification of EEG-based winking signals: a transfer learning and random forest pipeline. PeerJ.

[ref-14] Pappalardo A (2014). How dow you judge a skateboard contest?. http://www.jenkemmag.com/home/2014/04/07/how-do-you-judge-a-skateboard-contest/.

[ref-15] Qassim YT, Cutmore T, James D, Rowlands D (2012). FPGA implementation of Morlet continuous wavelet transform for EEG analysis.

[ref-16] Rangasamy K, As’ari MA, Rahmad NA, Ghazali NF (2020). Hockey activity recognition using pre-trained deep learning model. ICT Express.

[ref-17] Sokolova M, Lapalme G (2009). A systematic analysis of performance measures for classification tasks. Information Processing and Management.

[ref-18] Stein M, Janetzko H, Lamprecht A, Breitkreutz T, Zimmermann P, Goldlücke B, Schreck T, Andrienko G, Grossniklaus M, Keim DA (2018). Bring it to the pitch: combining video and movement data to enhance team sport analysis. IEEE Transactions on Visualization and Computer Graphics.

[ref-19] Türk Ö, Özerdem MS (2019). Epilepsy detection by using scalogram based convolutional neural network from EEG signals. Brain Sciences.

[ref-20] Vijay Anand S, Shantha Selvakumari R (2019). Noninvasive method of epileptic detection using DWT and generalized regression neural network. Soft Computing.

